# Augmenting Aquaculture Efficiency through Involutional Neural Networks and Self-Attention for Oplegnathus Punctatus Feeding Intensity Classification from Log Mel Spectrograms

**DOI:** 10.3390/ani14111690

**Published:** 2024-06-05

**Authors:** Usama Iqbal, Daoliang Li, Zhuangzhuang Du, Muhammad Akhter, Zohaib Mushtaq, Muhammad Farrukh Qureshi, Hafiz Abbad Ur Rehman

**Affiliations:** 1 National Innovation Center for Digital Fishery, Beijing 100083, China; usamaiqbal@cau.edu.cn; 2 Key Laboratory of Smart Farming Technologies for Aquatic Animal and Livestock, Ministry of Agriculture and Rural Affairs, Beijing 100083, China; 3 Beijing Engineering and Technology Research Center for Internet of Things in Agriculture, Beijing 100083, China; dzz643@cau.edu.cn; 4 College of Information and Electrical Engineering, China Agricultural University, Beijing 100083, China; muhammadakhter58@gmail.com; 5 Department of Electrical, Electronics and Computer Systems, University of Sargodha, Sargodha 40100, Pakistan; zohaib.mushtaq@uos.edu.pk; 6 Department of Electrical and Computer Engineering, Riphah International University, Islamabad 44000, Pakistan; muhammad.farrukh@riphah.edu.pk; 7 School of Engineering, University of Guelph, Guelph, ON N1G 2W1, Canada; habbadur@uoguelph.ca

**Keywords:** fish feeding behavior, spectrogram-based feature fusion, Discrete Wavelet Transform, Gabor filter, Local Binary Pattern, Laplacian High Pass Filter, temporal segmentation, sustainable resource management

## Abstract

**Simple Summary:**

Managing fish feeding well is important for both making fish farming better and keeping aquatic environments healthy. By looking at the sounds fish make, this study suggests a new way to learn about how they eat. We turn these sounds into pictures and use advanced computer methods to figure out the different ways people eat. Our method uses a strong deep learning model that can correctly group the eating habits of a certain type of fish, which helps us figure out how much and how often they eat. With a 97% success rate, this method shows a lot of promise for better running fish farms and protecting marine ecosystems. In the future, researchers might be able to add more types of data to this method, which could give us even more information about how to farm fish sustainably and manage ecosystems.

**Abstract:**

Understanding the feeding dynamics of aquatic animals is crucial for aquaculture optimization and ecosystem management. This paper proposes a novel framework for analyzing fish feeding behavior based on a fusion of spectrogram-extracted features and deep learning architecture. Raw audio waveforms are first transformed into Log Mel Spectrograms, and a fusion of features such as the Discrete Wavelet Transform, the Gabor filter, the Local Binary Pattern, and the Laplacian High Pass Filter, followed by a well-adapted deep model, is proposed to capture crucial spectral and spectral information that can help distinguish between the various forms of fish feeding behavior. The Involutional Neural Network (INN)-based deep learning model is used for classification, achieving an accuracy of up to 97% across various temporal segments. The proposed methodology is shown to be effective in accurately classifying the feeding intensities of *Oplegnathus punctatus*, enabling insights pertinent to aquaculture enhancement and ecosystem management. Future work may include additional feature extraction modalities and multi-modal data integration to further our understanding and contribute towards the sustainable management of marine resources.

## 1. Introduction

The *Oplegnathus punctatus* has high economic value because of its fast growth rate, low breeding cost, high survival rate, and high nutritional value [1]. This species is of significant commercial value in aquaculture, with unique feeding behaviors that make it a compelling study subject. It also serves as a model organism for optimizing feeding aquaculture practices. In recent years, it has become an economically important fish species for farming in China. Aquaculture encompasses more than just fish, including other aquatic organisms, such as shellfish and crustaceans, which play an essential role in addressing the ever-increasing global demand for seafood [2]. The optimal feeding of marine organisms in aquacultures plays a significant role in nutritional management and the growth rate [3]. The challenge of optimally feeding aquatic organisms arises from their varied metabolic demands and the difficulties in feeding various species a sufficient amount of feed to avoid underfeeding or overfeeding [4]. Underfeeding leads to slow growth and weakens their immunity against diseases, leading to poor health and a reduced growth rate [5]. Malnutrition and failures to thrive result in reduced yields and the growth of commercial aquaculture systems. In contrast, overfeeding leads to low feed efficiency, high concentrations of organic matter, and the proliferation of harmful bacteria that decrease water quality [5]. All of these factors reduce fish health and diminish the sustainability of aquaculture commercial systems [6].

Deep learning models have already been used in environmental sound classification [7,8], image classification [9,10], signal classification [11,12], and many medical applications [13,14,15]. Deep learning techniques have been used in aquaculture to address the problems of overfeeding and underfeeding, while simultaneously creating an efficient feeding system [16]. The ability of deep learning models to identify complex patterns from vast datasets presents an opportunity to improve aquaculture farms.

Traditionally, aquaculture utilized manual observation approaches, timed feeding, and nutritionally balanced feeds [17]. However, these techniques are not flexible enough to be made sustainable and result in underfeeding or overfeeding [18]. Given these impacts, it is evident that controlling fish growth and well-being by investing in intelligent feeding is a necessary practice to maintain profitable and sustainable aquaculture. The economic relevance and flexibility of *O. punctatus* make it a suitable species for studying feeding responses and optimizing growth in aquaculture. Our research intends to apply its unique properties to develop new strategies for enhancing feeding effectiveness and promoting growth in aquaculture environments. We have utilized deep learning to recognize feeding responses, i.e., observable behaviors associated with feeding activities, such as surface movements, acoustic signals, or visual aids, and our results demonstrate the potential of this approach in optimizing fish growth, feeding efficiency, and overall well-being in commercial aquaculture environments. This paper examines how deep learning can enhance the productivity, profitability, and well-being of *O. punctatus* in controlled aquaculture environments with a low computational architecture while striving for high performance.

## 2. Literature Review

The growth, health, and sustainability of aquaculture depend on fish-feeding behavior [16,19]. Researchers can improve fish growth by studying their feeding behavior, while reducing waste and environmental impact. Various studies have applied deep learning techniques to investigate fish feeding habits. Du et al. [16] developed a novel MobileNetV3-SBSC model for analyzing fish feeding behaviors by utilizing a Mel spectrogram—which visually depicts audio frequencies mapped to the human-perceptible Mel scale—and its attributes to categorize three levels of feeding intensity: vigorous, moderate, and none. They achieved 85.9% accuracy, representing substantial progress toward using deep learning to decode aquaculture-related actions. Cui et al. [19] leveraged Mel Spectrogram features and a convolutional neural network to classify fish feeding behaviors, obtaining a mean average precision of 74%. This measure considers both precision and recall rather than mere accuracy. Ubina et al. [20] assessed fish feeding intensity and employed a three-dimensional convolutional neural network alongside Optical Flow Frames, which visually portray the motion of objects between successive images or video frames by calculating the direction and magnitude of pixel or regional movement. Their model achieved an accuracy of 95%, demonstrating the utilization of advanced deep learning architectures in distinguishing highly complex behaviors in aquaculture systems.

Zhou et al. [21] utilized a CNN with computer vision techniques for fish feeding behavior analysis. CNN was used to detect saliency in images in real-time. A multi-crop voting strategy was used to classify feeding states in Nile tilapia. Their approach was able to classify feeding behaviors with an accuracy of 90%, showing the continued importance of integrating traditional computer vision methods with a deep learning approach to create systems that can robustly analyze behavior.

Similarly, the authors of the LC-GhostNet model, Du et al. [22], presented another fish feeding behavior analysis system, which introduces a new Feature Fusion Strategy, i.e., a technique that integrates multiple feature sets to create a more comprehensive data representation. Their model achieved an accuracy of 97.941%. They represent a notable improvement in fish feeding behavior analysis as their results show the potential of more sophisticated deep learning architectures for precise behavioral analyses in aquaculture. Zhang et al. [23] proposed the MSIF-MobileNetV3 model for analyzing fish feeding behavior through multi-scale information fusion (MSIF). The authors obtained an accuracy of 96.4%. This study reflects a recent trend of incorporating complex attention mechanisms into deep learning architectures for behavioral analysis tasks. Zhang et al. [24] created a MobileNetV2-SENet-based accounting method to identify feeding behavior in fish farms. The model achieved an accuracy of 97.76%. The authors demonstrated the feasibility of using pre-trained models and attention mechanisms to accurately classify complex behaviors in aquaculture. Yang et al. [25] developed a Dual Attention Network using Efficientnet-B2 to analyze short-term feeding behavior in fish farms. Their model achieved an accuracy of 89.56%. Although this accuracy is lower than in other similar studies, the authors argued that integrating attention mechanisms and efficient network architectures was shown to be critical for precise behavioral tasks.

Feng et al. [26] developed a lightweight 3D ResNet-GloRe network to classify fish feeding intensity in real-time video streams. Their model outperforms traditional 3D ResNet networks, achieving 92.68% classification accuracy. It also showcased the feasibility of incorporating relational reasoning modules for precise behavioral examination in aquaculture video streams. In their investigation, Zeng et al. [27] utilized acoustic signals and an improved Swin Transformer model to research fish feeding behaviors. Their Audio Spectrum Swin Transformer model, or ASST, achieved an accuracy of 96.16%, highlighting the effectiveness of attention mechanisms and spectrogram-based qualities for significantly accurate behavioral analysis in aquaculture. Meanwhile, Kong et al. [28] established a recurrent system using energetic learning to investigate fish feeding states. By combining the VGG16 network with lively learning strategies, their model accomplished 98% accuracy, requiring few labeled examples typically necessary for supervised coaching. This highlights the potential of lively learning approaches for productively categorizing behaviors in aquaculture with minimal supervised training data. Hu et al. [29] developed a fish feeding system using computer vision techniques. Instead of relying on underwater image analysis, their system utilized deep learning models to recognize the size of water waves caused by fish feeding, achieving an accuracy of 93.2% and providing an alternative solution for real-world aquacultures. Zheng et al. [30] focused on analyzing golden pompano school feeding behavior using a spatiotemporal analysis of a STAN (spectral Temporal Attention Network) to an aquaculture feeding behavior in aquaculture. The model achieved test results that outperformed all other proposed alternatives and subsequently demonstrated the efficiency of combining both temporal and spectral features to conduct highly precise behavioral analysis in fish in aquaculture tanks. Jayasundara et al. [31] introduced deep learning automatic fish grading using image-based quality assessment. The FishNET-S and FishNET-T model intelligently assessed quality in images for a series of grading use cases, reaching model testing accuracies of up to 84.1%. The analysis of these studies are summarized in Table 1.

## 3. Motivation and Significant Contributions

While various deep-learning architectures have been adopted for fish behavior analysis, we see a gap in the use of lightweight models. While many lightweight models like MobileNet demonstrate high accuracy, they may not be optimized for real-time analysis in resource-constrained environments. In aquaculture, edge devices often have limited computational power and memory, necessitating models that are not only lightweight but also maintain high accuracy. Furthermore, traditional lightweight models may not be tailored to the unique demands of fish-feeding behavior analysis, where the data are derived from acoustic signals rather than conventional image-based sources. This requires specialized feature extraction and handling, which many existing lightweight networks may not address adequately. Many of the existing models incur high computational costs and inference times, rendering them impractical for real-time deployment in aquaculture, while some studies adopt feature extraction techniques, and there is a lack of effort to explore state-of-the-art feature extraction such as how to perform feature extraction tailored specifically for audio-based analysis (e.g., Log Mel Spectrogram extraction). Many of the existing methods are likely not to satisfy the fish farmers’ requirement of low computation costs and inference times, especially if the objective is to deploy the model in aquaculture habitats. There is a gap in creating models specifically optimized for efficiency without compromising accuracy.

In this paper, a novel Involutional Neural Network (INN) [32] architecture was developed for fish behavior analysis in aquaculture. Unlike traditional Convolutional Neural Networks (CNNs), which use fixed kernels to extract features, INNs employ learnable filters that adapt based on context and allow for more flexible feature extraction. This adaptability makes INNs efficient in terms of computational resources and ideal for deployment in environments with limited capacity. Compared to transformers, which focus on self-attention mechanisms for capturing long-range dependencies, INNs maintain a focus on local features with a simpler structure, resulting in a more lightweight architecture. The proposed architecture is of a reduced size and exhibits significantly lower computational costs and faster inference times compared to traditional models for this application. Therefore, due to simplicity and speed, the developed architecture aims to tackle the identified research gaps by proposing Involutional Neural Network (INN) architecture tailored for reduced size and computational efficiency.

The significant contributions of this work are as follows:

- The utilization of Involutional Neural Network architecture for fish behavior analysis.
- We employ Log Mel Spectrograms converted from audio waveforms and subsequent feature extraction methods from Log Mel Spectrograms.
- Our proposed method prioritizes efficiency without compromising accuracy, making it suitable for real-time deployment in aquaculture environments.

The rest of the paper is structured as follows: Section 3 presents the dataset utilized and details of the Involutional Neural Network architecture. Section 4 provides results and discusses the findings, while Section 5 concludes the paper.

## 4. Materials and Methods

### 4.1. Experimental Setup and Materials

**Figure 1:**
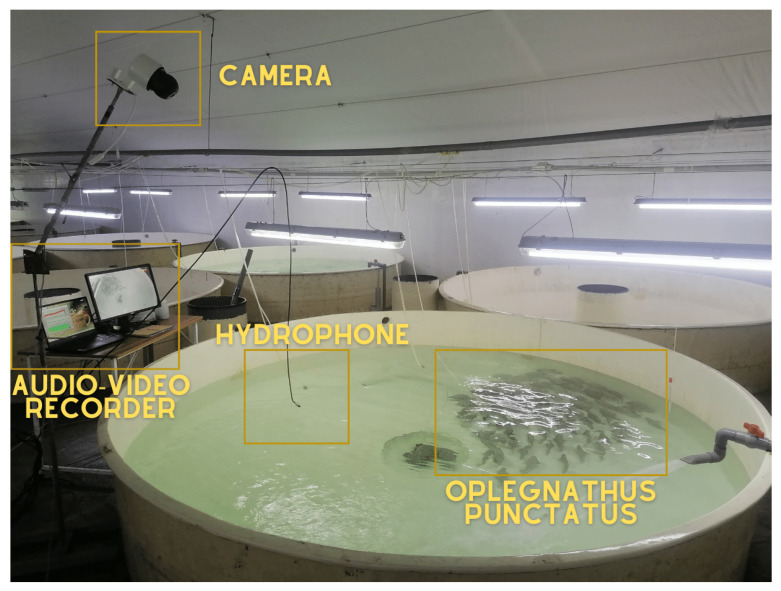
The structure of the experimental system of recirculating aquaculture.

Our experimental facility was established within the cutting-edge labs of Mingbo Aquatic Co., Ltd., situated in Yantai, Shandong Province, China. Figure 1 depicts the comprehensive apparatus employed in our investigation. This system integrated a Recirculating Aquaculture System (RAS), a battery of video capture technologies, and leading-edge acoustic data collection instruments to ensure thorough information aggregation. The RAS included culture tanks measuring 330 cm in diameter and 60 cm in depth. Within this system, environmental parameters were tightly regulated, such as maintaining temperature at precisely 26 ± 1 °C, dissolved oxygen levels exceeding 5.5 mg/L, a pH equilibrium of 7.2 ± 0.5, nitrate levels under 0.5 mg/L, and ammonia concentrations capped at ≤0.8 mg/L. The RAS was outfitted with an aeration unit, lighting fixtures, and a sophisticated water purification system to provide optimal circumstances for the *O. punctatus*.

As shown in Figure 1, the experimental setup accommodated five culture tanks, each dedicated to rearing varying numbers of fish—5, 15, 40, 70, and 100 individuals. This diversity in population size permitted the investigation of fish feeding practices under assorted conditions. Our video acquisition system employed a Hikvision (City Of Industry, CA, USA) color camera (model: DS-2SC3Q140MY-TE) with 4MP resolution and a frame rate of 25 fps. This assemblage was augmented with an IEEE802.3af standard POE switch (model: DS-3E0105P-E/M) and a Hikvision video recorder (model: DS-7104 N-F1) to guarantee high-quality video material throughout the experiment.

Concurrently, the acoustic data collection system incorporated an omnidirectional LST-DH01 digital hydrophone, exhibiting an operating frequency range of 1 Hz–100 kHz, with a sensitivity of 209 ± 1.5 dB. This hydrophone offered horizontal and vertical directivity of ±2 dB @ 100 kHz, 360° and a maximum sampling rate of 256KSPS. The auditory component of our information aggregation was further strengthened by a Legion Y7000 computer, equipped with an Intel Core i7–10750-H processor and 16 GB of RAM.

During the experiment, the *O. punctatus* were nourished with Santong Bio-engineering (Weifang, China) Co., Ltd.’s Sea Boy UFO series feed. The nutritional composition of this feed comprised a crude protein content of ≥48.0%, crude fat of ≥9.0%, lysine of ≥2.5%, and total phosphorus of ≥1.5%. The pellet size was standardized at 5 mm. Throughout the experiment, the feeding was was carried out in the exact location to maintain a constant distance from both the camera and the hydrophone. It was provided by experienced aquaculture technicians. Before the experiment, around 230 fish with an average mass of 200 ± 50 g underwent a month-long acclimatization period within the experimental system.

#### 4.1.1. Audio and Video Acquisition

Video data acquisition was executed precisely using the Hikvision camera positioned above the rearing tank, capturing comprehensive scene information. Operating at a rate of 25 frames per second, the camera recorded images, subsequently saving them to the video recorder. This process ensured a detailed temporal record of the fish within the rearing tank.

**Figure 2:**
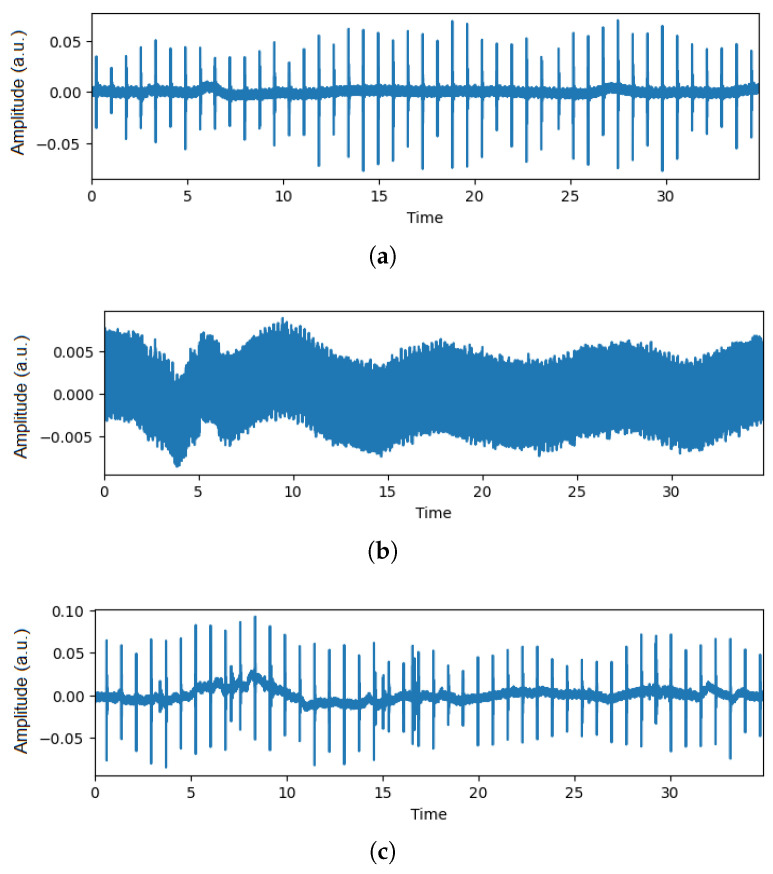
Audio waveforms captured from aquaculture: (**a**) Audio of None class, (**b**) Audio of Medium class, and (**c**) Audio of Strong class.

Simultaneously, audio data acquisition focused on capturing the sounds associated with fish feeding. The recording process transpired at a sample rate of 256 kHz. Fish were fed twice daily at 7:30 a.m. and 3:30 p.m., adhering to established guidelines for feeding in realistic aquaculture production environments. The feeding volume equated to 1.5% of the fish’s weight. Audio acquisition commenced 5 min before each feeding session and extended 5 min post-feeding, ensuring a comprehensive audio dataset that encapsulated the entire feeding event. The start and stop times for the feeding events were based on the fish’s activity level, indicating when feeding behavior began and ended. Figure 2 shows the audio waveforms captured during the different feeding intensities. It can be seen that the maximum amplitude goes up to 0.05 a.u. for the ‘None’ class, 0.007 a.u. for the ’Medium’ class, and 0.30 a.u. for the ‘Strong’ class. To illustrate the behavior of each class in acoustic signals, the y-axis was adjusted based on the maximum amplitude.

#### 4.1.2. Audio and Video Preprocessing

The collected dataset comprised 160 audio data samples gathered over 16 days during active fish feeding. Sophisticated tools such as Hikvision’s VSPlayer for video handling and Adobe Audition for audio handling were employed to synchronize the audio streams with corresponding video clips. This effort was undertaken to ensure an accurate assessment of feeding dynamics.

It was important to confirm that the audio streams were synchronized with matching video clips to guarantee precise timeline alignment. We were able to build a reliable foundation for examining feeding behavior by using this process, allowing us to more precisely identify instances of feeding and categorize them with enhanced precision.

Following the established feeding intensity standards in aquaculture [27] and drawing on expertise from experienced aquaculture technicians, the feeding intensity classifications were decided. The audio data were separated into three groups depending on the strength of feeding: “strong,” “moderate,” and “none.” The description of these classifications is displayed in Table 2.

Using the synchronized audio, audio segments matching each feeding intensity classification (which included “strong”, “moderate”, and “none”) were extracted. These segments were arranged relating to the strength and timeline that corresponded to where they were categorized in the video. Through employing this extraction process, it was confirmed that every single audio segment precisely represented the particular level of feeding strength.

To consider the variations in the strength of feeding over time, each audio clip was subsequently divided into periods of three seconds, four seconds, and five seconds. Through the process of timeline segmentation, a wide range of feeding behavior patterns were obtained, resulting in the establishment of a comprehensive database suitable for training and evaluating deep learning models. The amplitude of the signals from all three segmented classes were normalized to ensure consistency in signal magnitude representation. This dataset was created by selecting training and testing samples from each time category using a random selection of audio clips. The 80–20 format was used to select the samples. For three-second audio clips, 8055 audio clips were used for training and 1151 audio clips were used for testing. Similarly, 8123 audio clips were used for training and 1161 audio clips for testing for four-seconds audio clips. Additionally, 8234 audio clips were used for training and 1177 audio clips were used for testing for five-second audio clips.

### 4.2. Proposed Approach

In the pursuit of exploring the complex patterns within the audio data, an important step involved the transformation of raw audio waveforms into Log Mel Spectrogram images. This process holds significance in terms of (a) capturing the overall complex frequency characteristics of the fish feeding sounds, and (b) the representation of one-dimensional data to two-dimensional data as spectrogram images.

#### 4.2.1. Log Mel Spectrogram

A Mel spectrogram is a representation of the audio signal in the Mel frequency domain, offering a perceptually relevant visualization of the signal’s frequency components [7]. It plays a significant role in speech and audio processing, providing a more human-centric representation of sound frequencies [8].

The transformation from raw audio waveforms to Mel spectrograms involves several steps. The Mel scale, a perceptual scale of pitches approximating the human ear’s response to different frequencies, is utilized to convert the linear frequency scale into a logarithmic Mel scale. This conversion is achieved through the following formula [12]:

(1) $$Mel\left(f\left(t\right)\right)=2595\times \log_{10}\left(1+\frac{f(t)}{700}\right)$$

Here, f(t) represents the linear frequency in hertz.

Following the Mel scale conversion, the audio signal is divided into small overlapping frames, and a Fast Fourier Transform (FFT) is applied to each frame to obtain the magnitude spectrum. The resulting spectrum is then passed through a filter bank designed according to the Mel scale. This process effectively captures the energy distribution across several frequency bands in a more perceptually relevant manner.

The Log Mel Spectrogram equation can be expressed as [33]:

(2) $$S\left(f,t\right)=\log\sum_{m=1}^{M}H_{m}\left(f\right)\cdot {\left|X_{m}\left(t\right)\right|}^{2}$$

where:

- S(f,t) is the Mel spectrogram magnitude at frequency *f* and time *t*,
- $H_{m}\left(f\right)$ represents the filter bank response for the *m*-th Mel filter at frequency *f* in Mel filter bank M(f,t),
- $X_{m}\left(t\right)$ denotes the magnitude spectrum of the audio signal in the *m*-th Mel filter at time *t*.

The Mel filter bank M(f,t) [34] is a collection of bandpass filters that linearly transforms the frequencies into the logarithmic Mel scale. Each filter in the bank has a different frequency range, and the response is the sum of applying these filters to the signal; this shows how much energy or power is present in each frequency band. Mel filter banks can be computed from Equation (3):

(3) $$M\left(f,t\right)=\left\{\begin{array}{cc} \frac{f\left(t\right)-f_{c}\left(t-1\right)}{f_{c}\left(t\right)-f_{c}\left(t-1\right)} & \mathrm{for}\;f_{c}\left(t-1\right)\leq f\left(t\right)<f_{c}\left(t\right)\\ \frac{f\left(t\right)-f_{c}\left(t+1\right)}{f_{c}\left(t\right)-f_{c}\left(t+1\right)} & \mathrm{for}\;f_{c}\left(t\right)\leq f\left(t\right)<f_{c}\left(t+1\right)\\ 0 & \mathrm{others}. \end{array}\right.$$

where f(t) is linear frequency and $f_{c}\left(t\right)=t\cdot \delta Mel\left(f\right)$ are center frequencies on the Mel-scale.

**Figure 3:**
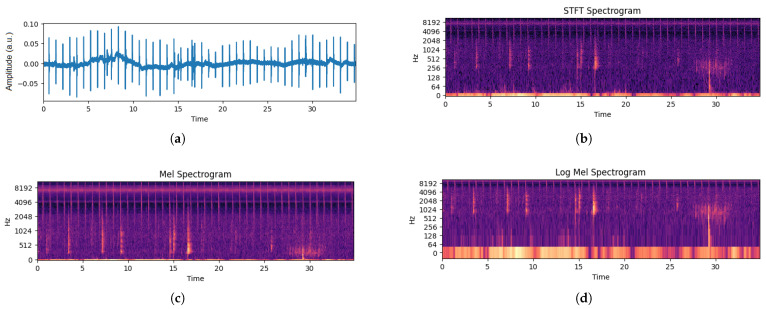
Conversion of audio waveforms to Log Mel Spectrograms (using magma colormap): (**a**) audio waveform, (**b**) corresponding short-term Fourier Transform, (**c**) Mel spectrogram, and (**d**) Log Mel Spectrogram.

**Figure 4:**
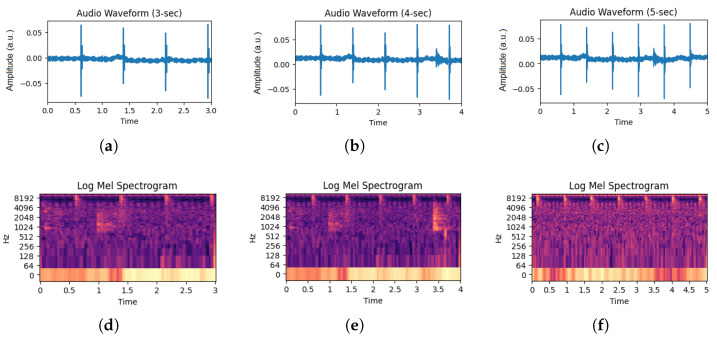
Segmentation of audio waveforms and their corresponding Log Mel Spectrograms (using magma colormap): (**a**) Audio of None class, (**b**) Audio of Medium class, (**c**) Audio of Strong class, (**d**) Log Mel Spectrogram of None class, (**e**) Log Mel Spectrogram of Medium class, and (**f**) Log Mel Spectrogram of Strong class.

The Log Mel Spectrogram (LMS) encapsulates frequency information, providing a foundation for subsequent feature extraction and model training. The LMS allows for a representation of the acoustic complexities and classification of the associated feeding intensities in *O. punctatus* aquaculture. Figure 3 depicts the process of converting an audio sample into an LMS spectrogram image. Figure 4 illustrates samples from each class in Log Mel Spectrogram images. Both of these figures use magma colormap.

The next step involves extracting distinctive features from the LMS. The selected features are the Discrete Wavelet Transform (DWT) [35], Gabor filter [36], Local Binary Pattern (LBP) [37], and Laplacian high-pass filter [38]. The DWT provides a comprehensive multi-scale analysis for identifying patterns across different frequency bands, thus resulting in differentiating feeding intensities [39]. Gabor filters enhance the local frequency features, aiding in the detection of specific modulation frequencies that correspond to different feeding behaviors [40]. The LBP captures local texture information, which helps in recognizing subtle differences in the spectrogram, making it valuable for detailed acoustic analysis [41]. The Laplacian high-pass filter highlights significant transitions in the frequency content, enhancing the visibility of important features for fish-feeding behavior classification [42]. A combined image was created by the fusion of all of these features.

#### 4.2.2. Discrete Wavelet Transform (DWT)

The Discrete Wavelet Transform (DWT) [35] represents a powerful tool for extracting both time and frequency information from signals. In the context of the LMS, the DWT can decompose the signal into different frequency components, allowing for the analysis of signal patterns at various scales [39]. This multiscale analysis is crucial for identifying unique features in acoustic signals, which is useful in classification tasks. Applied to the Mel spectrogram S(f,t), the DWT decomposes the image into Approximate and Detail coefficients across different scales. The mathematical expression for the DWT can be defined as:

(4) $$DWT\left(S\left(f,t\right)\right)=\sum_{i=1}^{N}\sum_{j=1}^{M}S_{i}\left(f,t\right)\cdot \psi_{i,j}\left(t\right)$$

Here, $S_{i}\left(f,t\right)$ and $\psi_{i,j}\left(t\right)$ represent the scale and wavelet functions, respectively.

The 2D Discrete Wavelet Transform (DWT) is applied to the Mel spectrogram S(f,t) to decompose it into approximation and detail coefficients. The transformation is performed in both the frequency *f* and time *t* dimensions. The 2D DWT coefficients $W_{\varphi }\left(j_{0},k\right)$ and $W_{\psi }\left(j,k\right)$ are computed using:

(5) $$\begin{array}{cc} W_{\varphi }\left(j_{0},k\right) & =\frac{1}{\sqrt{M}}\sum_{x}S\left(x,t\right)\varphi_{j_{0},k}\left(t\right) \end{array}$$

(6) $$\begin{array}{cc} W_{\psi }\left(j,k\right) & =\frac{1}{\sqrt{M}}\sum_{x}S\left(x,t\right)\psi_{j,k}\left(t\right) \end{array}$$

Here, $\varphi_{j_{0},k}\left(t\right)$ and $\psi_{j,k}\left(t\right)$ are the scaling and wavelet functions, respectively. At level 3 of the decomposition, the detail coefficients $W_{\psi }\left(j,k\right)$ are set to zero, effectively removing the high-frequency components and denoising the signal. The denoised coefficients are denoted as $W_{\psi }^{\mathrm{denoised}}\left(j,k\right)$. The denoised coefficients, along with the approximation coefficients $W_{\varphi }\left(j_{0},k\right)$, are used to reconstruct the synthesized image using the Inverse 2D DWT (IDWT) operation and are given by:

(7) $$\begin{array}{cc} S_{\mathrm{DWT}}\left(f,t\right)= & \frac{1}{\sqrt{M}}\sum_{k}W_{\varphi }\left(j_{0},k\right)\varphi_{j_{0},k}\left(f\right)+\\ \; & \frac{1}{\sqrt{M}}\sum_{j=j_{0}}^{\infty }\sum_{k}W_{\psi }^{\mathrm{DWT}}\left(\;j,k\right)\psi_{j,k}\left(f,t\right) \end{array}$$

**Figure 5:**
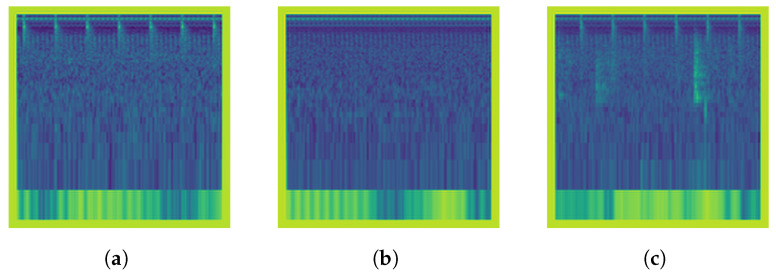
Discrete wavelet transform extracted from the corresponding Log Mel Spectrogram Images: (**a**) None, (**b**) Medium, and (**c**) Strong.

This reconstructed denoised image $S_{\mathrm{DWT}}\left(f,t\right)$ represents the Mel spectrogram with the high-frequency noise removed, enhancing the clarity of the underlying features. The samples from each DWT extracted features are illustrated in Figure 5. The process is expressed in Algorithm 1.
**Algorithm 1** Denoising the Mel Spectrogram image using DWT**Input:** Mel spectrogram S(f,t)Apply 2D-DWT to S(f,t) and obtain coefficients $W_{\varphi }\left(j_{0},k\right)$ and $W_{\psi }\left(j,k\right)$**Level 3 Decomposition:****for** j=1 to $j_{0}$ **do**    **for** *k* in the range of coefficients at level 3 **do**        $W_{\psi }\left(j,k\right)\leftarrow 0$       ▹ Remove high-frequency components    **end for****end for**Reconstruct denoised coefficients: $W_{\psi }^{\mathrm{DWT}}\left(j,k\right)$Reconstruct synthesized image:$S_{\mathrm{DWT}}\left(f,t\right)\leftarrow \sum_{k}\frac{W_{\varphi }\left(j_{0},k\right)\varphi_{j_{0},k}\left(f\right)}{\sqrt{M}}+\sum_{j=j_{0}}^{\infty }\sum_{k}\frac{W_{\psi }^{\mathrm{DWT}}\left(j,k\right)\psi_{j,k}\left(f,t\right)}{\sqrt{M}}$**Output:** Denoised Mel spectrogram $S_{\mathrm{DWT}}\left(f,t\right)$

#### 4.2.3. Gabor Filter

Gabor filters can extract features related to the modulation frequencies of the signal. This technique is effective in enhancing the local frequency features, thus aiding in the differentiation of complex acoustic patterns [40]. The Gabor filter [36] is defined in the spectral domain as:

(8) $$G\left(x,y;\lambda ,\theta ,\psi ,\sigma ,\gamma \right)=\exp\left(-\frac{x^{\prime 2}+\gamma^{2}y^{\prime 2}}{2\sigma^{2}}\right)\cdot \cos\left(2\pi \frac{x^{\prime }}{\lambda }+\psi \right)$$

Here, $x^{\prime }$ and $y^{\prime }$ represent the rotated and scaled coordinates, λ is the Wavelength of the sinusoidal factor, θ is the orientation of the Gabor filter, ψ is the phase offse, σ is the standard deviation of the Gaussian envelope, and γ is the spectral aspect ratio. The application of the Gabor filter to the Mel spectrogram S(f,t) is $S_{Gabor}\left(f,t\right)$ and is expressed as:

(9) $$S_{\mathrm{Gabor}}\left(f,t\right)=S\left(f,t\right)*G\left(f,t;\lambda ,\theta ,\psi ,\sigma ,\gamma \right)$$

And the application of the Gabor filter to the Mel spectrogram can be represented as:

(10) $$S_{\mathrm{Gabor}}\left(f,t\right)=S\left(f,t\right)*\exp\left(-\frac{f^{\prime 2}+\gamma^{2}t^{\prime 2}}{2\sigma^{2}}\right)\cdot \cos\left(2\pi \frac{f^{\prime }}{\lambda }+\psi \right)$$

**Figure 6:**
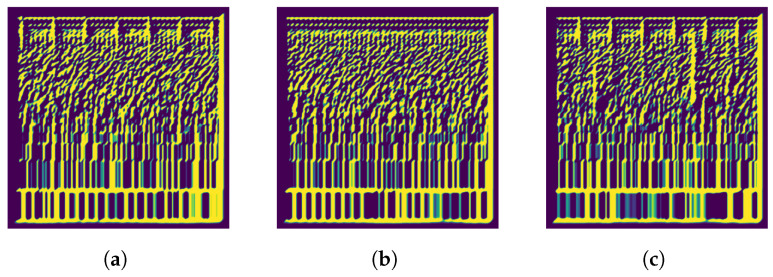
Gabor filter applied on Log Mel Spectrogram Images of each class: (**a**) None, (**b**) Medium, and (**c**) Strong.

This operation will yield the Mel spectrogram after being filtered by the Gabor filter with the specified parameters. In this study, we set the parameters as: λ=30, θ=π/4, ψ=0, σ=4, and γ=0.5. These parameters were selected after a series of experiments in which we tested a range of values to determine the optimal settings. We aimed to identify parameter values that would provide distinct differences among the three feeding intensity classes: “Strong”, “Medium”, and “None” as illsutarated in Figure 6.

#### 4.2.4. Local Binary Pattern

The Local Binary Pattern (LBP) [37] operator captures local patterns within the Mel spectrogram. The LBP can be used to capture local texture patterns, which are critical for recognizing subtle differences in acoustic signals [41]. The application of the LBP to the Mel spectrogram image S(f,t) is expressed as:

(11) $$S_{LBP}\left(f,t\right)=\sum_{i=0}^{7}2^{i}\cdot \delta \left(S\left(f_{i},t_{i}\right)-S\left(f,t\right)\right)$$

**Figure 7:**
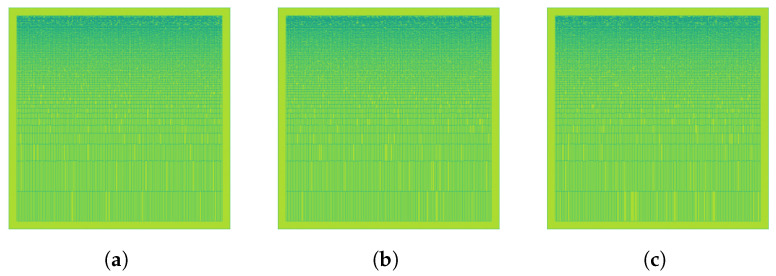
Local Binary Pattern extracted from Log Mel Spectrogram Images of each class: (**a**) None, (**b**) Medium, and (**c**) Strong.

Here, $S(f_{i},t_{i})$ represents the intensity value of the neighboring pixel, and S(f,t) is the intensity of the center pixel at coordinates f,t. Samples from each class are presented in Figure 7.

#### 4.2.5. Laplacian High Pass Filter

The Laplacian High Pass Filter (LHPF) [38] is applied to the Mel spectrogram image S(f,t) to enhance fine details and edges. When applied to the LMS, it highlights the rapid changes in the frequency content, making it easier to identify significant transitions in the signal. This is particularly useful for enhancing the contrast of the LMS image and emphasizing the important features for classification tasks [42]. The operation of the Laplacian High Pass Filter involves subtracting a smoothed version of the image from the original image and can be expressed as:

(12) $$\begin{array}{c} S_{\mathrm{LHPF}}\left(f,t\right)=S\left(f,t\right)-\left(\sum_{i,j}G\left(i,j,\sigma \right)\cdot S\left(f-i,t-j\right)\right.\\ \left.+\left(\frac{\partial^{2}S\left(f,t\right)}{\partial f^{2}}+\frac{\partial^{2}S\left(f,t\right)}{\partial t^{2}}\right)\right) \end{array}$$

**Figure 8:**
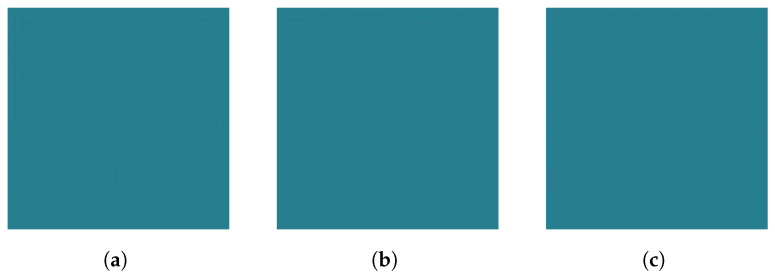
Laplacian High Pass filter applied on Log Mel Spectrogram Images of each class: (**a**) None, (**b**) Medium, and (**c**) Strong.

Here, the $S_{\mathrm{LHPF}}\left(f,t\right)$ is the Mel spectrogram after processing; S(f,t) is the original Mel spectrogram; G(i,j,σ) is the 2D Gaussian kernel with standard deviation σ; and $\frac{\partial^{2}S\left(f,t\right)}{\partial f^{2}}$ and $\frac{\partial^{2}S\left(f,t\right)}{\partial x^{2}}$ represent the second partial derivatives with respect to *f* and *t*, respectively. Samples from each class are illustrated in Figure 8.

#### 4.2.6. Combination of Extracted Features

**Figure 9:**
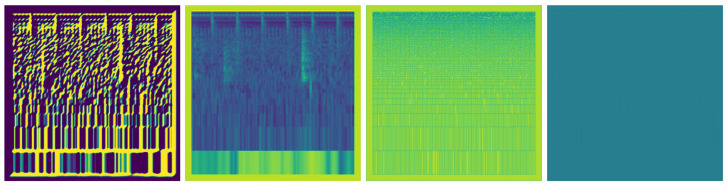
Combined Features extracted from LMS for a sample of ’Strong’ class as an input to the model.

Finally, the images resulting from each feature extraction method are horizontally concatenated to form a composite image as illustrated in Figure 9. This combination of distinctive features presents a holistic representation, capturing complex details of the *O. punctatus* feeding intensity in a unified format. This feature set, which contains both temporal and spectral information for comprehending fish feeding dynamics, is used as the input for later model training and analysis. The process is illustrated in Algorithm 2.
**Algorithm 2** Preprocessing of the Log Mel Spectrogram image and resulting feature image**Input:** Mel spectrogram image S(f,t)**Output:** Concatenated processed image $S_{\mathrm{Processed}}\left(f,t\right)$**Step 1: Denoising with DWT**$S_{DWT}\left(f,t\right)\leftarrow \mathrm{Apply}\;\mathrm{DWT}\left(S\left(f,t\right),\;\mathrm{Level}=3,\mathrm{Wavelet}=\mathrm{db}10\right)$**Step 2: Filtering with Gabor filter**$S_{Gabor}\left(f,t\right)\leftarrow \mathrm{Apply}\;\mathrm{GaborFilter}\left(S\left(f,t\right),\lambda =30,\theta =\pi /4,\psi =0,\sigma =4,\gamma =0.5\right)$**Step 3: Operation with LBP**$S_{LBP}\left(f,t\right)\leftarrow \mathrm{Apply}\;\mathrm{LBP}\left(S\left(f,t\right),P=8,R=1,\mathrm{Method}=\mathrm{Uniform}\right)$**Step 4: Filtering with Laplacian High Pass Filter (LHPF)**$S_{LHPF}\left(f,t\right)\leftarrow \mathrm{Apply}\;\mathrm{LHPF}\left(S\left(f,t\right)\right)$**Step 5: Concatenation**$S_{\mathrm{Processed}}\left(f,t\right)\leftarrow \mathrm{Concatenate}\left(S_{DWT}\left(f,t\right),S_{Gabor}\left(f,t\right),S_{LBP}\left(f,t\right),S_{LHPF}\left(f,t\right)\right)$**Return** $S_{\mathrm{Processed}}\left(f,t\right)$

### 4.3. Network Architecture

In our experiments, we used an Involutional Neural Network (INN) with self-attention. The INN is a novel architecture that utilizes involution layers to effectively capture both local and global features for the purpose of classification. This architecture has been purposefully designed to have a small number of parameters in order to make it deployable on edge devices that have a limited number of computational resources.

#### 4.3.1. Involutional Layer with Self-Attention

One of the key components that contributes to the perception of the high-performance of the involution network is the mechanism of the involution layer, which replaces the traditional convolutional layers [32]. Through the process of learning dynamic receptive fields for each output vector position, the involution layer becomes more flexible, allowing it to adapt to a variety of contexts for feature extraction. Convolutional neural networks are used to generate feature maps by applying a set of learnable filters to the input image. The operation of the involution layer on the input feature $S_{Processed}\left(f,t\right)$, where *f* represents the spectral frequency and *t* represents the time, is presented in the following manner:

(13) $$\begin{array}{c} S_{\mathrm{scaled}}\left(i,j\right)=\frac{1}{K_{1}\times L_{1}}\sum_{m=0}^{K_{1}-1}\sum_{n=0}^{L_{1}-1}\\ S_{Processed}\left(f\times \mathrm{stride}+m,t\times \mathrm{stride}+n\right) \end{array}$$

(14) $$\begin{array}{cc} Z_{1}\left(i,j\right)= & \sum_{m=0}^{0}\sum_{n=0}^{0}S_{\mathrm{scaled}}\left(i+m,j+n\right)\cdot W_{1}\left(m,n\right)+b_{1}\\ Z_{2}\left(i,j\right)= & \mathrm{ReLU}(Z_{1}\left(i,j\right))\\ Z_{3}\left(i,j\right)= & \sum_{m=0}^{K_{2}-1}\sum_{n=0}^{L_{2}-1}\mathrm{ReLU}\left((Z_{2}\left(i+m,j+n\right)\right)\cdot W_{2}\left(m,n\right)+b_{2}) \end{array}$$

Here, $Z(f^{\prime },t^{\prime })$ represents the output at position $\left(f^{\prime },t^{\prime }\right)$ in the feature map., s(f+m,t+n) denotes the input image values at position (f+m,t+n), W(m,n) is the weight corresponding to the convolutional filter at position (m,n), and *b* is the bias term. This convolutional operation is applied across the spectral dimensions of the input image. The parameters include the filter size (K×L) and the bias term *b*. The activation functions ReLU (f(x)=max(0,x)) are applied after the convolution to introduce non-linearity.

To further enhance the network’s capability to capture long-range dependencies, a self-attention mechanism is incorporated. This mechanism enables the model to weigh the importance of different spectral positions in the input, allowing it to focus on relevant regions while suppressing irrelevant information. The self-attention mechanism is mathematically expressed as:

(15) $$\begin{array}{cc} F(x) & =\mathrm{reshape}(W_{2}\left(m,n\right),\left(K_{2}\times L_{2},1\right))\\ G(x) & =\mathrm{reshape}(W_{2}\left(m,n\right),\left(K_{2}\times L_{2},1\right))\\ H(x) & =\mathrm{Reshaped}\;\mathrm{Input}\;\mathrm{Patches}\\ Att & =\mathrm{Dropoutsoftmax}\left(\frac{F\left(x\right)\cdot G\left(x\right)\cdot H{\left(x\right)}^{T}}{\sqrt{d_{k}}}\right)\cdot H\left(x\right)\\ O & =Att\cdot H(x) \end{array}$$

Here, F(x), G(x), and H(x) represent the query, key, and value matrices, respectively, and $d_{k}$ is the dimensionality of the critical vectors.

#### 4.3.2. Involutional Neural Network Architecture

**Figure 10:**
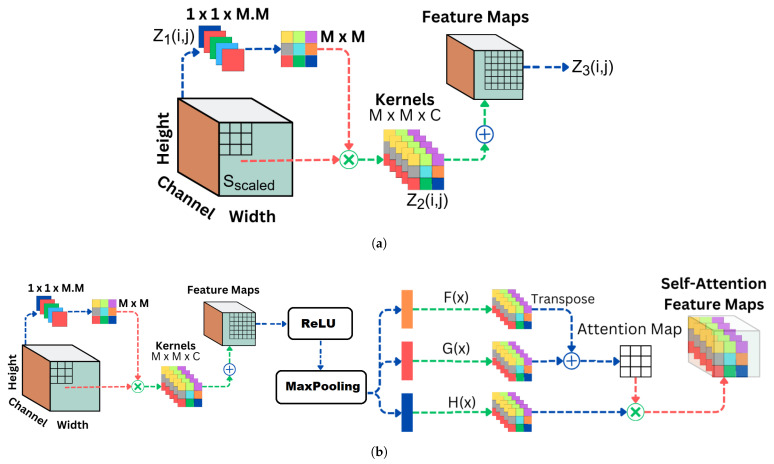
Involutional Neural Network: (**a**) involution layer, (**b**) involution layer with self-attention.

The overall architecture of the INN with self-attention involves stacking multiple involution layers with intermittent self-attention modules. The images were fed to the INN with a size of 224×224×3. A rescaling layer of range (0,1) was also introduced. In INN, the *Adam* optimizer was used, the total number of epochs was 50, the batch size was 32 uniformly, the Rectified Linear Unit (ReLU) activation function was used for the first four layers, and the Softmax activation function was utilized for the final layer. The general architecture of INN implementation is depicted in Figure 10. The parameters of the filters, the strided max-pooling function, and the receptive field are described in the following:

- Layer 1: The first layer of the model consists of involution operations, as shown in Figure 10a. It has three output channels and uses a dynamic kernel of size 3×3. The stride is set to 1, and the reduction ratio is 2. Following the involution operation, a max-pooling layer with a pool size of 2×2 is applied, and ReLU activation is used.
- Layer 2: Similar to the first layer, the second layer also uses involution operations with similar parameters. It uses a 3×3 dynamic kernel and has three output channels. The reduction ratio is 2, and the stride is set to 1. ReLU activation comes after the involution operation, and a max-pooling layer with a pool size of 2×2 is applied.
- Layer 3: The third layer continues with involution operations. It has three output channels and uses a dynamic kernel of size 3×3. The stride is set to 1, and the reduction ratio is 2. After the involution operation, a max-pooling layer with a pool size of 2×2 is applied, followed by ReLU activation.
- Layer 4: The fourth layer reshapes the output of the third layer into a format suitable for further processing. It reshapes the output into ‘(height × width, 3)’.
- Layer 5: The fifth layer applies a self-attention mechanism to capture dependencies between different patches of the feature map as illustrated in Figure 10b. It uses an attention width of 15 and a sigmoid activation function.
- Layer 6: Following the self-attention layer, the output is flattened to prepare it for the fully connected layers.
- Layer 7: The sixth layer consists of a fully connected dense layer with 32 hidden units and ReLU activation.
- Layer 8: The final layer is another fully connected dense layer with three output units, corresponding to the number of classes in the dataset. It does not have an explicit activation function as it is followed by the softmax activation applied during model compilation for probabilistic classification.

The summary of the proposed model is given in Table 3.

The model is trained using the processed Mel spectrogram images as input and the corresponding feeding intensity labels. Training involves minimizing the categorical cross-entropy loss function to ensure the model accurately predicts the feeding intensity. The loss function is given as:

(16) $$L=-\frac{1}{N}\sum_{i=1}^{N}\sum_{k=1}^{3}y_{i,k}\cdot \log\left({\hat{y}}_{i,k}\right),$$

where *N* represents the total number of samples; $y_{i,k}$ is the one-hot encoded ground truth for sample *i*; and class *k*, ${\hat{y}}_{i,k}$ is the predicted probability for sample *i* and class *k*.

### 4.4. Performance Evaluation Metrics

Performance Evaluation Metrics for multi-class classification such as Accuracy, Macro Weighted Precision (MWP), Macro Weighted Recall (MWR), and Macro F1-score have been used.

Accuracy for multi-class classification is given as:

(17) $$Accuracy=\frac{TP+TN}{TP+TN+FP+FN}$$

Precision and recall for binary-class classification for a generic class *k* are given as:

(18) $$\begin{array}{cc} Precision_{k} & =\frac{TP_{k}}{TP_{k}+FP_{k}} \end{array}$$

(19) $$\begin{array}{cc} Recall_{k} & =\frac{TP_{k}}{TP_{k}+FN_{k}} \end{array}$$

For multi-class classification, MWP and MWR are used and given as:

(20) $$\begin{array}{cc} MWP & =\frac{\sum_{k=1}^{K}Precision_{k}}{K} \end{array}$$

(21) $$\begin{array}{cc} MWR & =\frac{\sum_{k=1}^{K}Recall_{k}}{K} \end{array}$$

The Macro F1-score for multi-class classification is given as:

(22) $$Macro\;F1\text{-}score=2\times \left(\frac{MWP\times MWR}{MWP^{-1}+MWR^{-1}}\right)$$

## 5. Results and Discussion

### 5.1. Results and Comparison

We distributed the dataset into three sub-datasets, with each representing a different duration of audio segments: 3 s, 4 s, and 5 s. Each sub-dataset was split into training and testing sets in an 80–20 ratio to ensure robust evaluation of the model’s performance. We utilized the proposed Involutional Neural Network (INN). On dataset of 3-s dataset, the INN achieved an accuracy of 97.11%. On a dataset of 4-s, the model achieved an accuracy of 92.73%, while on a dataset of 5-s, the model achieved an accuracy of 90.56%. Table 4 shows the performance achieved by the INN on each dataset.

**Figure A1:**
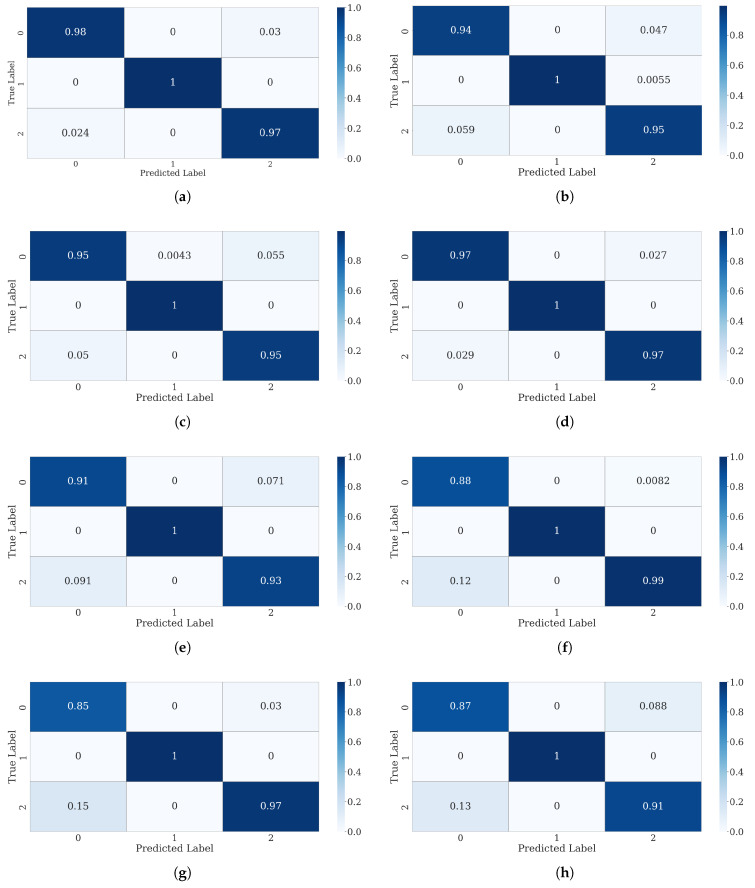
Confusion matrices: (**a**) Involutional Neural Network, (**b**) VGG16, (**c**) VGG19, (**d**) ResNet50, (**e**) Xception, (**f**) EfficinetNet-b0, (**g**) InceptionNetV3, and (**h**) MobileNetV2.

Since the model achieved better accuracy on a 5-s dataset, we have utilized several pre-trained transfer learning models for comparison. Figure A1 illustrates the confusion matrices of the proposed model and the pre-trained models. Although the confusion matrices do not illustrate a significant difference among the models, a closer look at parameters provides a different outlook. Table 5 illustrates the performance observations of various deep learning models, including the proposed Involutional Neural Network (INN), alongside well-established architectures such as VGG16, VGG19, ResNet50, Xception, EfficientNet-B0, InceptionV3, and MobileNetV2.

As observed, the proposed INN demonstrates a substantially smaller model size and number of parameters compared to variant counterparts. This feature makes it a strong deployment candidate in resource-constrained environments, especially in edge computing, where memory and computational resources are more expensive. The lightweight architecture of the INN also manifests excellent classification accuracy, outperforming several established models with significantly larger parameter counts—a strong indication of its capability to encapsulate intricate patterns within audio features and accurately classifying feeding intensities. Moreover, the efficient training and inference times of the INN highlighted its potential suitability in real-time applications, where quick predictions are crucial in ensuring timely decision-making processes.

VGG16 and VGG19 demonstrate higher parameter counts and larger model sizes compared to the INN despite their simplicity and effectiveness, which, while achieving reasonable accuracies, quantify slower training and inference times and computational overhead, which are less attractive in edge computing scenarios; ResNet50 and Xception also show competitive accuracies yet much slower training and inference times and computational overhead compared to the INN. The EfficientNet-B0, however, demonstrates an interesting trade-off between model complexity and accuracy. Having fewer parameters and a smaller model size compared to a few of its counterparts, the EfficientNet-B0 still managed to demonstrate commendable accuracy (competing closely with the VGG19) and relatively faster training and inference times, making it a strong choice for applications that require a balance between model performance and computational efficiency.

InceptionV3 and MobileNetV2 manifest distinct characteristics in their parameter counts, model sizes, and performance metrics; the former demonstrates a competitive number of parameters and model sizes compared to ResNet50 and Xception, showcasing near-competitive accuracies and slightly slower training and inference times, which may limit its potential suitability in real-time applications, specifically, compared to the model performance and computational efficiency of the MobileNetV2. The latter has a considerably lower number of parameters and model sizes compared to its counterparts in this comparison, as well as demonstrating impressive accuracies near the competitive level of accuracies exhibited by more complex architectures; moreover, its notably faster training and inference times make it a strong deployment candidate in latency-sensitive application, where real-time predictions are crucial.

### 5.2. Involutional Neural Networks Compared with Convolutional Neural Networks

Involutional Neural Networks (INNs) differ from traditional Convolutional Neural Networks (CNNs) primarily in how they process input data. Instead of using fixed convolutional kernels to extract features from spectral images, INNs apply involution operations, which involve learnable filter kernels that can adapt to the context of the data. Thus, they flexibly capture local and contextual features related to our dynamic local feature approach. Since our task differs from conventional image-based applications in which CNNs excel at identifying spatial patterns, here we use Mel spectrograms, which represent audio signals in time and frequency dimensions. The structural differences of spectrograms from standard spectral images and their non-spatial nature require an anatomy to capture patterns there. The INN uses more local resupply learning that focuses on the feature extraction’s temporal and frequency characteristics. Considering how Mel spectrograms are dramatically different from the natural form of spectral images, the INN adapts its receptive domain to the surrounding context to extract meaningful features from time-frequency data. With fewer parameters and low computation costs compared to standard CNNs, the INN provides our light feature extraction method that facilitates processing Mel spectrograms. This lightness is a more significant advantage when deploying models on the brink or in surging-platform situations. The implementation of the INN in our experiment is effective due to its flexibility and efficiency of feature extraction from Mel spectrograms. This is critical in pattern recognition and distinguishing the survival time and intensity levels. The INN is resilient enough to handle tasks due to its adaptivity and simplicity, which diverges from the standard spectral images. However, it is less promoted when exposed to performance issues than complex integrative tasks. When given a broader dataset, the model capacity may shrink due to its simplicity and reduced sets of parameters. Major models prefer complex architectures and higher sets of parameters, which provide a shorthand, detailed feature increase in speed and accuracy with leveling. While the INN is efficient, CNNs would perform better when deployed in more enormous datasets tasks or broader complex set tasks.

### 5.3. Potential Deployment of INN Model on Edge Devices

As edge devices usually have constrained computational resources, the model architecture chosen has a strong influence regarding performance, efficiency, and viability in real-world use-cases. This is especially relevant when considering the proposed implementation of the INN model on edge devices. The implementation strategy for the INN is framing the choice of model in providing a lightweight and efficient model that can be supported in a low-resources environment like edge devices. INNs were developed to maintain high accuracy and effectiveness while reducing the model’s parameters and computational overhead. The INN parameters were kept to a minimum in order to reduce computational overhead while maintaining high accuracy, whereas traditional Convolutional Neural Networks can be resource intensive. INNs have optimized parameters and accuracy, making them efficient. The cost savings from INNs over other CNN-based methods with edge devices come from less model complexity. The convolutional parameters are higher than INNs; this requires less memory capacity because the computational cost during the inference process is lower. Since in most cases the devices are power-limited, the INN model is suitable. Furthermore, the INN uses involution operations over traditional convolutions. Since the computational cost of the latter is more significant but with involution, the computation is less; hence there is less inference time. This is vital for real-time-based edge devices. Although the exact savings depend on various factors, such as the device specification and the model architecture, an overall reduction in resource requirement and processing time is achieved with the INN, hence making it suitable as a lightweight model for an edge network as opposed to a bigger CNN-based model with large resource requirements. The savings might not be sufficient to make an impact, but with computational efficiency even small differences are significant. The traditional CNN-based method requires complex operations and more memory capacity, leading to long inference time and power consideration. This makes INNs a viable source based on deployment area. For instance, an aquaculture monitoring system and real-time data analysis are instances that may require this.

## 6. Conclusions

This paper presents a novel utilization of the Log Mel Spectrogram features that introduces a holistic approach for identifying fish feeding dynamics. It includes four methods of feature extraction: the Discrete Wavelet Transform, the Gabor filter, the Local Binary Pattern, and the Laplacian High Pass Filter. The Log Mel Spectrogram allows one to obtain a deeper insight into the fish’s feeding behavior. Moreover, it leverages a novel model, the Involutional Neural Network with self-attention capabilities. As a result, the model can efficiently capture the token relationship and self-attention in the obtained feature space. Consequently, the model has a distinguishable performance verified on the different duration intervals, demonstrating high accuracy, precision, recall, and F1-score. The introduced methodology can distinguish between three feeding intensities of *O. punctatus*, hence revealing evidence about its feeding behavior. As for future work, there are many potential directions to pursue, including integrating more techniques for feature extraction, working with multimodal datasets, and building more sophisticated deep learning models. Involutional Neural Networks have proven to be efficient, simplifying the structure, computation, and number of parameters of the model relative to Convolutional Neural Networks, which can deal with large datasets. By simplifying the network, we reduce its complexity and performance. Convolutional Neural Networks possess more complex architectures and parameter counts and thus are beneficial for large datasets and complicated inquiries because they possess higher accuracy and generalization. Based on this, it is safe to say that it is plausible to deploy INNs to the edge considering all the requirements. This approach can achieve a balance between performance and competence.

## Figures and Tables

**Table 1:** Analysis of earlier studies on fish feeding behavior using deep learning.

Reference	Year	Technique/Model	Classes	Accuracy
[16]	2023	MobileNetV3-SBSC with Mel Spectrogram	Strong, Medium, and None	85.9%
[19]	2022	CNN with Mel Spectrogram	None, Weak, Medium, and Strong	74% (mAP)
[20]	2021	3D CNN with Optical Flow Frames	None, Weak, Medium, and Strong	95%
[21]	2019	CNN with Machine Vision	-	90%
[22]	2024	LC-GhostNet with Feature Fusion Strategy	Strong, Medium, Weak, and None	97.941%
[23]	2023	MSIF-MobileNetV3	-	96.4%
[24]	2022	MobileNetV2-SENet	-	97.76%
[25]	2021	Dual attention network with Efficientnet-B2	-	89.56%
[26]	2022	3D ResNet-GloRe network	-	92.68%
[27]	2023	ASST with improved Swin Transformer	Strong, Medium, Weak, and None	96.16%
[28]	2022	VGG16 with Active Learning	-	98%
[29]	2022	Computer Vision for Fish Feeding	-	93.2%
[30]	2023	Spatiotemporal Attention Network (STAN)	-	97.97%
[31]	2023	FishNET-S and FishNET-T	-	84.1%, 68.3%

**Table 2:** The classification standards for fish feeding activity.

Feeding Activity	Descriptions
Strong	Fish move freely between food items and consume all the available food.
Medium	Fish move to take food but return to their original positions.
None	Fish do not respond to food.

**Table 3:** Summary of the proposed Involutional Neural Network.

Layer	Output Shape	Param #
InputLayer	[(None, 224, 224, 3)]	0
Rescaling	(None, 224, 224, 3)	0
Involution	((None, 224, 224, 3),	26
	(None, 224, 224, 9, 1, 1))	
ReLU	(None, 224, 224, 3)	0
MaxPooling2D	(None, 112, 112, 3)	0
Involution	((None, 112, 112, 3),	26
	(None, 112, 112, 9, 1, 1))	
ReLU	(None, 112, 112, 3)	0
MaxPooling2D	(None, 56, 56, 3)	0
Involution	((None, 56, 56, 3),	26
	(None, 56, 56, 9, 1, 1))	
ReLU	(None, 56, 56, 3)	0
MaxPooling2D	(None, 28, 28, 3)	0
Reshape	(None, 784, 3)	0
SeqSelfAttention	(None, 784, 3)	257
Flatten	(None, 2352)	0
Dense	(None, 32)	75,296
Dense	(None, 32)	1056
Dense	(None, 3)	99
Trainable params: 76,780 (299.92 KB)		
Non-trainable params: 6 (24.00 Byte)		
Total params: 76,786 (299.95 KB)		

**Table 4:** Comparison of the performance of INN on sub-datasets.

Dataset	Accuracy	MWP	MWR	F1-Score
3-s	97.11%	97.10	97.10%	97.10%
4-s	92.73%	92.74	92.74%	92.74%
5-s	90.56%	90.53	90.53%	90.54%

**Table 5:** Comparison of the performance of Pretrained Transfer Learning Models on 5-s dataset.

Model	Parameters	Size	Accuracy	MWP	MWR	F1-Score	Training Time (s)	Inference Time (s)
VGG16	16,320,579	62.26 MB	95.84%	95.85%	95.85%	95.84%	509.127	2.551
VGG-19	21,630,275	82.51 MB	90.56%	90.53%	90.53%	90.53%	591.452	2.337
ResNet50	30,010,499	114.48 MB	97.86%	97.86%	97.88%	97.88%	424.957	1.711
Xception	27,284,267	104.08 MB	93.90%	93.89%	93.92%	93.92%	550.481	2.232
EfficientNet-B0	8,063,910	30.76 MB	95.40%	95.39%	95.39%	95.39%	320.851	1.236
InceptionNetV3	25,079,843	95.67 MB	93.26%	93.21%	93.22%	93.21%	340.652	1.403
MobileNet-V2	6,272,323	23.93 MB	91.97%	91.97%	91.97%	91.99%	216.605	0.884
**sProposed INN**	**76,786**	**299.95 KB**	**97.11%**	**97.10%**	**97.10%**	**97.10%**	**129.401**	**0.553**

## Data Availability

The datasets presented in this article are not readily available because the data are part of an ongoing study. Requests to access the datasets should be directed to corresponding author.
